# Lumbosacral epidural administration of 4% articaine with epinephrine in dromedary camels (*Camelus dromedarius*): anatomical and clinical evaluation

**DOI:** 10.3389/fvets.2026.1807758

**Published:** 2026-05-07

**Authors:** Tarik N. Misk, Adel Ibrahim Almubarak, Rasha Yassin Elkhidr, Abdelrahman M. A. Elseory, Razan Mamoun Naji, Sayed Fathi El-Hawari

**Affiliations:** 1 Department of Animal and Veterinary Sciences, College of Agricultural and Marine Sciences, Sultan Qaboos University, Muscat, Oman; 2 Department of Clinical Sciences, College of Veterinary Medicine, King Faisal University, Al-Ahsa, Saudi Arabia; 3 Department of Anatomy, College of Veterinary Medicine, King Faisal University, Al-Ahsa, Saudi Arabia; 4 College of Veterinary Medicine, King Faisal University, Al-Ahsa, Saudi Arabia

**Keywords:** articaine HCL, camels, epidural anesthesia, lumbosacral space, motor blockade

## Abstract

Epidural analgesia is commonly performed in camels via the first intercoccygeal space. However, the use of lumbosacral epidural injection has not been previously described in camels, and articaine hydrochloride (HCl) is rarely used in veterinary practice. This study was conducted to evaluate the efficacy of lumbosacral epidural administration of 4% articaine HCl with epinephrine in camels. An anatomical study was performed on six cadaveric trunks of male camels (age 5–10 years) to identify the depth, dorsal, and ventral width of the lumbosacral space. An anesthetic study was performed on six dromedary camels (three males and three females), aged 7–15 years and weighing 382 ± 152.4 kg. A 0.22 mg/kg dose of 4% articaine HCl with epinephrine (0.01 mg/mL) was administered epidurally. Analgesia and degree of ataxia were evaluated using numeric analgesic and ataxia scoring systems. The onset of analgesia and ataxia and the time to unaided standing after recovery were recorded. Cardiorespiratory parameters were also monitored. The spinal cord was located at a depth of 9.72 ± 0.58 cm from the skin. The dorsal length of the lumbosacral space was 9.32 ± 0.62 cm, while the ventral length was 4.33 ± 0.29 cm. Rapid onset of analgesia in the tail was observed at 4.6 ± 0.6 min. Recumbency began at 8.6 ± 2.7 min, and camels regained unaided standing after 228 ± 19 min. Alterations in heart rate and mean arterial blood pressure were recorded. Lumbosacral epidural administration of 4% articaine HCl with epinephrine in camels provided rapid onset, mild analgesia (median analgesic score 1–2) in the tail, perineal, umbilical, and flank regions; moderate analgesia in the inguinal region (median analgesic score 2–2.5); and moderately deep analgesia in the hind limbs (median analgesic score 2–3). This was accompanied by long-lasting recumbency and minimal clinically relevant cardiorespiratory changes.

## 1 Introduction

Camels are important animals because of their tolerance of tropical climates. The camel locomotor system is of particular importance because camels are used in racing sports (1). Camels are subjected to numerous surgical affections involving the tail, rectum, hind limb, pelvis, inguinal, and perineal regions (2–4).

Ruminants are generally poor candidates for general anesthesia due to the high risk of regurgitation and subsequent aspiration of ruminal contents or saliva into the lungs when the airway is unprotected. Furthermore, tympany was frequently noticed in ruminants during prolonged lateral recumbency associated with surgical procedures (5, 6). Consequently, regional anesthetic techniques are preferred in these species, most commonly achieved through perineural or epidural administration of local anesthetic agents. Among these techniques, caudal epidural analgesia is widely used to facilitate surgical interventions involving the perineum, rectum, and vagina in standing animals, with local anesthetics being the agents of choice (2).

The most common sites for epidural injection in camels are either the first intercoccygeal space or the sacrococcygeal space (7–9). In small animals, cattle, and small ruminants, lumbosacral epidural injection is frequently used to provide epidural analgesia (10, 11). Caudal intercoccygeal or sacrococcygeal epidural analgesia provides adequate anesthesia for the most caudal regions, including the tail, anus, and perineum. However, many surgical interventions in camels, such as hind-limb orthopedic surgeries, digit amputations, udder surgeries, castration, management of obstetrical disorders, treatment of gluteal wounds, and correction of preputial disorders, require more cranial lumbosacral analgesia. However, no previous studies have described the use of the lumbosacral space as a site for epidural injection in camels. Although specific morphometric data on the camel lumbosacral space are scarce, in other domestic species, such as cattle, horses, and dogs, the lumbosacral space is commonly used when a more cranial block is required using a smaller volume of anesthetic agent (12).

The amide structure of articaine is similar to that of other local anesthetics, but it contains an additional ester group that is quickly hydrolyzed by esterases, shortening its duration of action. Articaine has good lipid solubility (partition coefficient of 52) and low pKa (7.8) (13). Moreover, a distinctive structural feature of articaine is the presence of a thiophene ring instead of the typical benzene ring. This structural modification increases lipid solubility and membrane permeability, allowing improved diffusion through neural tissues. Articaine produces a rapid onset of action and successful local anesthesia. The blocking action of articaine on the sodium channel is state-dependent, with the highest affinity for the open state, an intermediate affinity for the inactivated state, and the lowest affinity for the resting state (13). Its pharmacological properties make it a potential candidate for epidural and regional analgesia in veterinary medicine. Due to its relatively high lipid solubility and efficient tissue diffusion, articaine is expected to provide rapid-onset epidural analgesia with moderate duration (as it has an ester group) that is quickly hydrolyzed by esterases (14). Compared with lidocaine, articaine demonstrates similar pharmacodynamic characteristics but may provide a slightly faster onset and somewhat longer duration of action in certain regional techniques. This difference may be explained by articaine’s dual metabolic pathway, which involves hydrolysis by plasma esterases and hepatic metabolism, leading to rapid systemic clearance while maintaining effective local anesthetic activity (15). From a safety perspective, articaine is rapidly metabolized by nonspecific plasma esterases, which reduces the risk of systemic accumulation. However, as with other local anesthetics used in neuraxial techniques, careful dosing and proper injection technique are essential to minimize potential complications, and careful monitoring remains critical for safe neuraxial use of articaine in veterinary practice (16). The addition of adrenaline at concentrations of 1:60,000, 1:100,000, or 1:200,000 reduces systemic absorption and prolongs the duration of anesthetic action (13, 17, 18). Recently, 4% articaine hydrochloride (HCl) with epinephrine has been used in veterinary practice for brachial plexus and pudendal nerve blocks in goats (19).

Accordingly, the present randomized study was designed to evaluate the efficacy of 4% articaine HCl with epinephrine as a novel epidural anesthetic agent in camels and to provide a detailed anatomical description of the lumbosacral space as a previously undescribed epidural injection site in this species. It was hypothesized that the lumbosacral approach would allow easier and more accurate epidural injection, and the use of 4% articaine HCl with epinephrine would produce potent and long-lasting analgesia.

## 2 Materials and methods

### 2.1 Anatomical description

This study was conducted on six male camel trunks (aged 5–10 years) obtained from the Al-Ahsa slaughterhouse, Saudi Arabia. Following slaughter and skinning, each fresh trunk cadaver was sectioned longitudinally along the median plane to expose the vertebral canal. The lumbosacral space was identified in the longitudinal section between the seventh lumbar (L7) and first sacral (S1) vertebrae. The depth from the superficial fascia to the spinal canal, the dorsal width between the spinous processes of L7–S1, and the ventral width at the level of the articular processes were measured at the midline using a calibrated digital caliper. All measurements were recorded in centimeters and tabulated for analysis.

### 2.2 Anesthetic evaluation

#### 2.2.1 Animals

This study was approved by the Deanship of Scientific Research Ethics Committee at King Faisal University (KFU-REC-2025-JAN-ETHICS2849). Six clinically healthy dromedary camels (three males and three females), aged 7–15 years and weighing 382 ± 152.4 kg, were randomly selected using Microsoft Excel. The animals were housed in pens, fed grass hay, and had free access to drinking water without fasting before anesthesia. All camels were dewormed one month before the start of the experiment. Two female camels were in the last trimester of pregnancy. Given the limited sample size, this study should be considered a preliminary study.

#### 2.2.2 Experimental procedure

**Figure 1:**
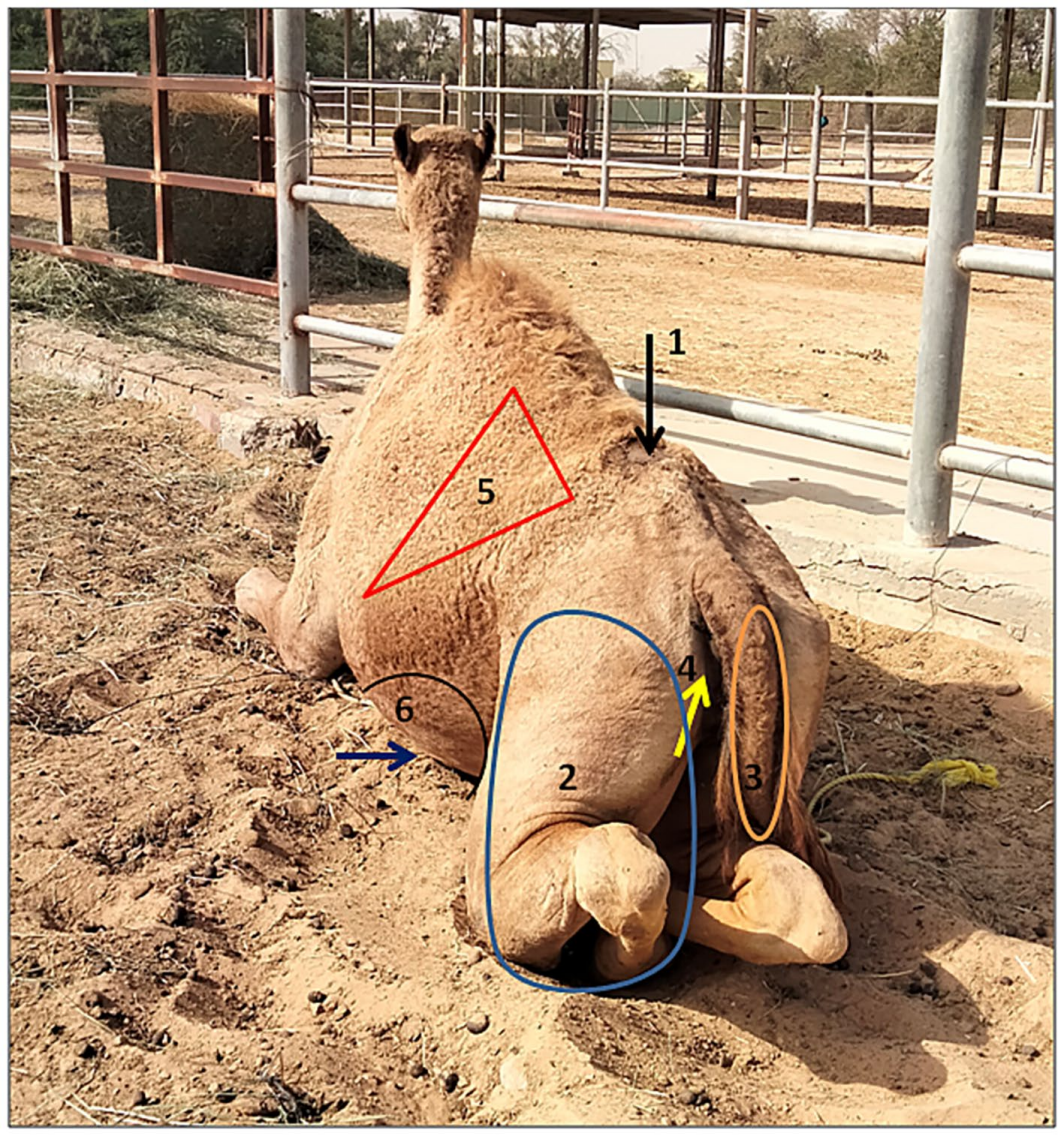
Demonstrated sites of lumbosacral epidural injection and different examined body regions after injection of articaine HCl with epinephrine in camels (1, site of lumbosacral epidural injection; 2, site of stimulus application in the hind limb; 3, site of stimulus application in the tail; 4, site of stimulus application in perineal region; 5, site of stimulus application in flank region; and 6, site of stimulus application in umbilical region).

Camels were restrained in sternal recumbency. The wings of the ilium were used as anatomical landmarks to locate the lumbosacral space (Figure 1). The injection site was clipped with electric clippers and aseptically prepared using an antiseptic scrub, and all procedures were performed under sterile conditions. To minimize muscular spasm during epidural needle placement and prevent needle displacement, 2 mL of the anesthetic solution (4% articaine HCl with epinephrine) was injected subcutaneously at the intended epidural site.

An 18-gauge, 9 cm spinal needle with a stylet was then introduced into the lumbosacral space. Correct needle placement was confirmed using the hanging drop technique and by the absence of resistance during injection. As previously described (20) for lidocaine epidural injection in camels, articaine HCl (4%; Septanest SP, Septodont, France) with epinephrine (0.01 mg/mL) was administered epidurally at a dose of 0.22 mg/kg over 1 min (zero time).

To standardize the injected volume and control anesthetic distribution within the spinal canal among camels of different body weights, the calculated dose was diluted with normal saline (0.9% sodium chloride; Pharmaceutical Solution Industry, Saudi Arabia). Saline was added to achieve a total injection volume of 0.03 mL/kg. This volume is somewhat close to the previously described volume in cattle (21). Following completion of the epidural injection, camels were released and allowed to walk freely within the yard.

#### 2.2.3 Assessment of analgesia

Before epidural injection (baseline), analgesia was assessed while camels were restrained in sternal recumbency using a numerical analgesic score modified from scores previously described in goats and sheep (5, 6) (Table 1). Analgesia was evaluated in the tail, perineum, inguinal, flank, umbilical, and hind limb regions (Figure 1). Following epidural injection, analgesia was periodically reassessed after the camels became staggered or recumbent using the towel forceps clamping method at 20, 30, 60, 90, 120, and 180 min in the same anatomical regions. The onset of analgesia and the time to the onset of hind limb struggling (onset of ataxia) were recorded. The time to recumbency and the time to unaided standing following full recovery were also documented.

**Table 1:** Description of analgesic score used for evaluation of anesthesia in different body regions after lumbosacral epidural injection of articaine HCL 4% epinephrine in camels.

Anti-nociception score	Description
0 (no analgesia)	Animal refuse stimulus and reacted strongly (head and limb movements, vocalization)
1 (mild analgesia)	Animal showed skin shivering and mild reaction against stimuli (forceps closed to the first ratchet)
2 (moderate analgesia)	Animal showed moderate reaction against clamping (forceps closed to the second ratchet)
3 (moderately deep analgesia)	Animal showed slight reaction against clamping (forceps closed to the third ratchet)
4 (deep analgesia)	Animal showed no reaction against complete clamping stimuli.

Ataxia scores were evaluated before and after epidural injection using a five-point numerical scale. A score of 0 indicated normal locomotion; a score of 1 indicated mild ataxia with slight stumbling while preserved ambulation; a score of 2 represented moderate ataxia with marked stumbling and obvious incoordination; a score of 3 denoted severe ataxia with inability to walk and dragging of the hind limbs; and a score of 4 indicated complete recumbency.

#### 2.2.4 Cardiorespiratory monitoring

Cardiovascular and respiratory parameters, including heart rate, arterial blood pressure, respiratory rate, and body temperature, were recorded at baseline (0 min) and at 20, 30, 60, 120, and 180 min following lumbosacral epidural injection. Heart rate was measured by auscultation with a stethoscope; arterial blood pressure and body temperature were measured using a multiparameter monitor (LifeVet; Eickemeyer Medizintechnik für Tierärzte KG, Tuttlingen, Germany); and respiratory rate was determined by observing chest wall excursions.

#### 2.2.5 Statistical analysis

Parametric data, including mean arterial blood pressure, heart rate, respiratory rate, and rectal temperature, were tested for normality using the Shapiro–Wilk test. Repeated measures ANOVA was used to assess the effect of anesthesia on each variable individually, with effect sizes reported as generalized eta squared (*η*^2^G). *Post hoc* paired t-tests with Holm–Bonferroni correction were used to compare different time points. Parametric data are presented with 95% confidence intervals.

Non-parametric data, including analgesic and ataxia scores, were analyzed using the Friedman test, with effect sizes reported as Kendall’s W. Post hoc Wilcoxon signed-rank tests with Holm–Bonferroni correction were used to compare different time points. Nonparametric data are presented as median (minimum-maximum). Statistical significance was set at *p* < 0.05.

## 3 Results

### 3.1 Anatomical description

**Figure 2:**
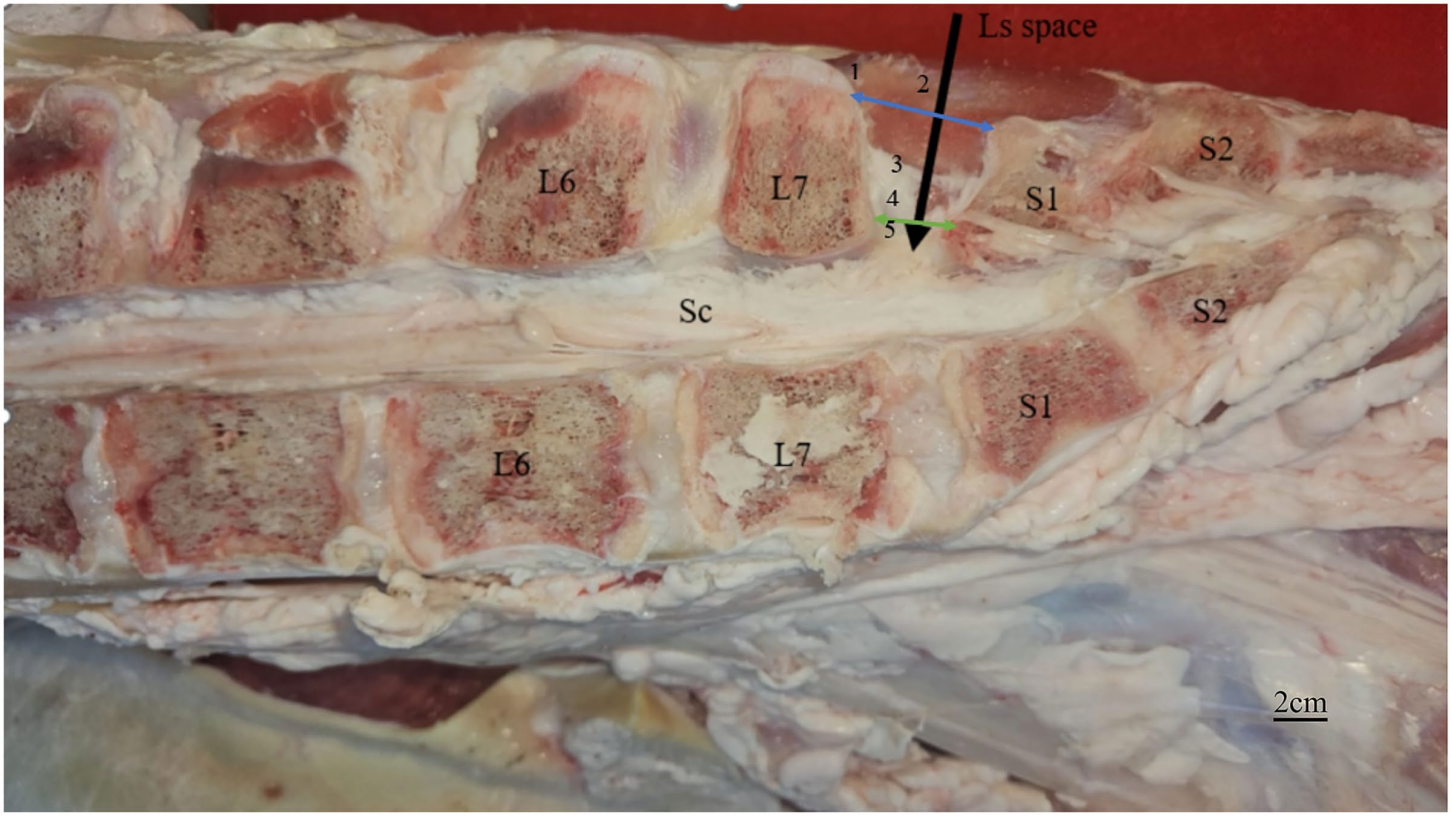
Anatomical description of the lumbosacral space in camel (black arrow), the dorsal distance between the spinous processes of the 7th lumbar vertebra and the 1st sacral vertebra (blue two head arrow), and the ventral length at the level of the articular processes of these vertebrae (green two head arrow). L7, 7th lumbar vertebra; L6, 6th lumbar vertebra; S1, 1st sacral vertebra; S2, 2nd sacral vertebra; Sc, spinal cord; 1, superficial fascia; 2, multifidus lumborum muscle; 3, supraspinous ligament; 4, interspinous ligament; 5, ligamentum flavum.

The camel lumbosacral junction site consisted of the skin, superficial fascia, multifidus lumborum muscle, supraspinous ligament, interspinous ligament, and ligamentum flavum, followed by the epidural space. The depth of the spinal cord from the skin was 9.72 ± 0.58 cm. The dorsal length between the spinous processes of the seventh lumbar (L7) and first sacral (S1) vertebrae was 9.32 ± 0.62 cm, whereas the ventral length at the level of the articular processes of these vertebrae measured 4.33 ± 0.29 cm (Figure 2).

### 3.2 Anesthetic evaluation

The onset of analgesia following lumbosacral epidural injection of 4% articaine HCl with epinephrine occurred at 4.6 ± 0.6 min in the tail, 7.6 ± 2.5 min in the perineal region, 7.6 ± 2.5 min in the inguinal region, 10.0 ± 0.5 min in the flank region, 6.7 ± 2.9 min in the umbilical region, and 5.6 ± 1.1 min in the hind limbs. The onset of ataxia was observed at 8.6 ± 2.7 min after epidural injection. Camels became recumbent at 21.0 ± 3.5 min post-injection and regained unaided standing after 228 ± 19 min, resulting in a total recumbency duration of 203 ± 19.8 min. Disappearance of analgesia was first observed in the tail and flank regions at 180 ± 48.9 min, followed by the umbilical region at 195 ± 57.4 min, the perineal region at 225 ± 57.4 min, the inguinal region at 240 ± 48.9 min, and finally the hind limbs at 255 ± 90 min.

**Figure 3:**
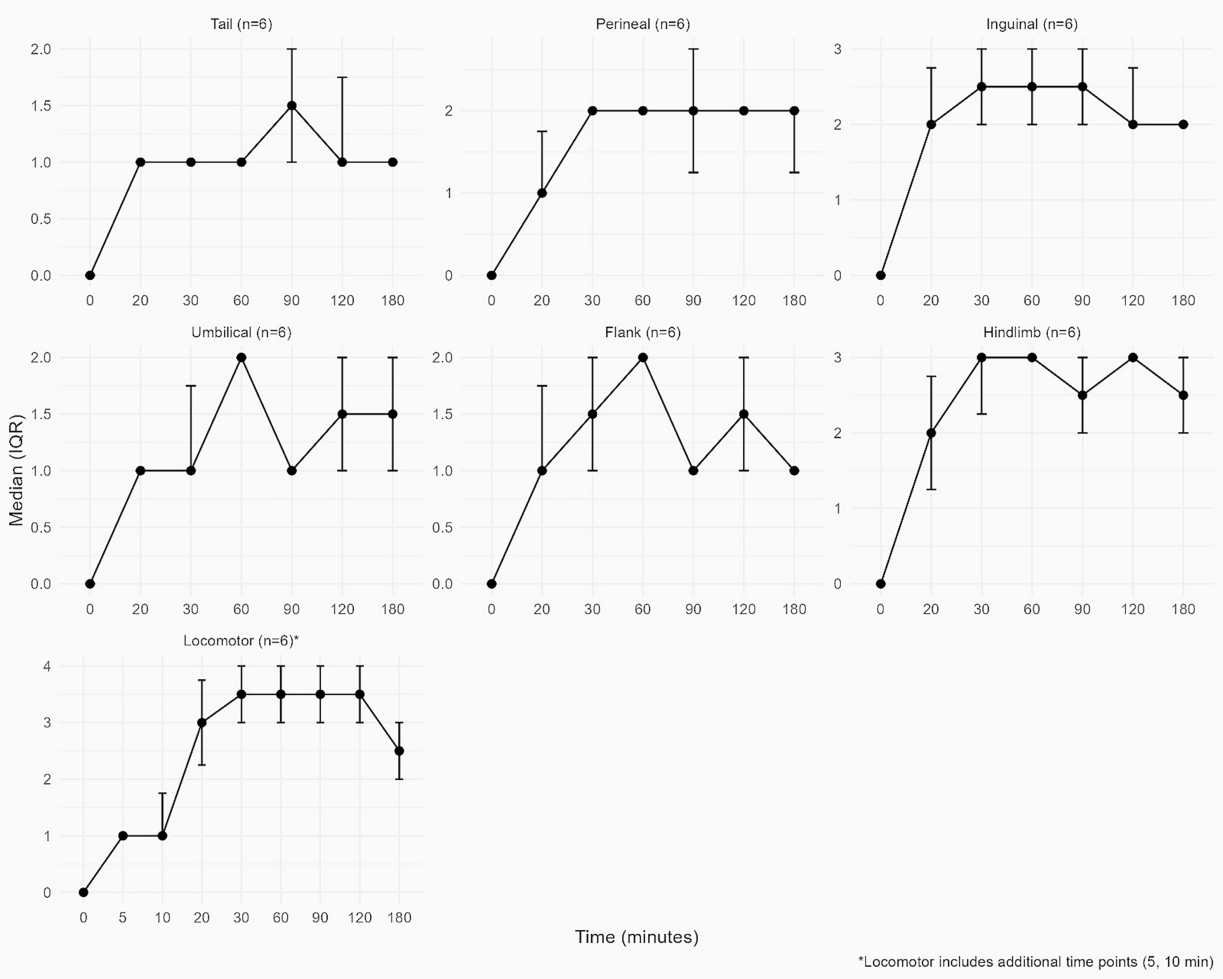
Analgesic score at different examined body regions (tail, perineal, inguinal, hind limb, umbilical, and flank) and locomotor (ataxia) score represented as median and interquartile range (IQR) at the baseline and at different time points after epidural injection of articaine HCl with epinephrine in camels.

Due to the small sample size of the examined camels (*n* = 6), some pairwise comparisons of analgesic and locomotor scores may not reach statistical significance after Holm–Bonferroni correction despite significant overall effects. Analgesic scores showed significant variation from baseline in all examined regions at all assessment times (Table 2). Maximal clinical analgesia of epidural injection of articaine HCl 4% with epinephrine appeared in the flank region (size effect = 0.629), whereas a lower effect appeared in the perineal region (size effect = 0.535). While the overall maximal clinical effect due to lumbosacral epidural injection of articaine HCl 4% with epinephrine in the present study appeared in the ataxia score (size effect = 0.84) (Table 2). The analgesic score is demonstrated in Figure 3. Ataxia score revealed a statistical and clinical deviation from the baseline, beginning at 5 min and persisting until 180 min following lumbosacral epidural injection of 4% articaine HCl with epinephrine. However, after the Holm–Bonferroni correction, no significant differences in analgesic or ataxia scores were detected across all different time points in all examined regions (Table 3).

**Table 2:** Illustrated Friedman test, effect size (Kendall W test) and descriptive statistics for non-parametric data (analgesic and ataxia scores) after lumbosacral epidural injection of articaine HCl 4% with epinephrine in camels.

Parameter	Analgesic score						Ataxia score
	Tail	Perineal	Inguinal	Umbilical	Flank	Hind limb	
*N*	6	6	6	6	6	6	6
*p*-value	0.001	0.004	0.003	0.001	0.001	0.002	2.97E-06
Kendall W	0.623	0.535	0.556	0.623	0.629	0.578	0.836937
Descriptive statistics (median, minimum–maximum)							
Baseline	0 (0–0)	0 (0–0)	0 (0–0)	0 (0–0)	0 (0–0)	0 (0–0)	0 (0–0)
5 min	ND	ND	ND	ND	ND	ND	1 (1–1)
10 min	ND	ND	ND	ND	ND	ND	1 (1–2)
20 min	1 (1–3)	1.5 (1–2)	2 (1–3)	1 (1–2)	1 (1–2)	2 (1–3)	3 (2–4)
30 min	1 (0–2)	2 (1–2)	2.5 (1–3)	1 (1–2)	1.5 (1–2)	3 (1–3)	3.5 (1–4)
60 min	1 (1–1)	2 (1–3)	2.5 (1–3)	2 (1–2)	2 (1–2)	3 (1–3)	3.5 (1–4)
90 min	1.5 (1–2)	2 (1–3)	2 (1–3)	1 (1–2)	1 (1–2)	3 (2–3)	3.5 (1–4)
120 min	1 (1–2)	2 (1–3)	2 (2–3)	1.5 (1–2)	1.5 (1–2)	3 (1–3)	3.5 (1–4)
180 min	1 (1–2)	2 (1–3)	2 (1–3)	1.5 (1–2)	1 (1–2)	3 (1–3)	2.5 (1–4)

ND, not detected.

**Table 3:** Results of Wilcoxon test after Holm–Bonferroni correction for non-parametric data (analgesic and ataxia scores) with multiple comparisons at different time points pre and post lumbosacral epidural injection of articaine HCL 4% with epinephrine in camels (non-adjusted significant data is the only presented data).

Tail region	*N*	*p*-value	Confidence indicator 95% (low)	Confidence indicator 95% (high)	Holm–Bonferroni corrected *p*-value
Baseline–20 min	6	0.026	−1	−1	0.552
Baseline–30 min	6	0.048	−1	−1	0.763
Baseline–60 min	6	0.026	−1	-1	0.552
Baseline–90 min	6	0.032	−2	−1	0.552
Baseline–120 min	6	0.03	−1.50004	−1	0.552
Baseline–180 min	6	0.026	−1	−1	0.552
Perineal region					
Baseline–20 min	6	0.03	−1.50004	−1	0.61
Baseline–30 min	6	0.026	−2	−2	0.552
Baseline–60 min	6	0.031	−2.99992	−1.00008	0.61
Baseline–90 min	6	0.034	−3	−1	0.61
Baseline–120 min	6	0.031	−2.99992	−1.00008	0.61
Baseline–180 min	6	0.034	−2.50004	−1	0.61
Inguinal region					
Baseline–20 min	6	0.034	−3	−1.49996	0.64
Baseline–30 min	6	0.034	−3	−2	0.64
Baseline–60 min	6	0.032	−3	−2	0.64
Baseline–90 min	6	0.032	−3	−2	0.64
Baseline–120 min	6	0.03	−2.50004	−2	0.64
Baseline–180 min	6	0.031	−2.99992	−1.00008	0.64
Umbilical region					
Baseline–20 min	6	0.026	−1	−1	0.526
Baseline–30 min	6	0.03	−1.50004	−1	0.549
Baseline–60 min	6	0.026	−2	−2	0.526
Baseline–90 min	6	0.02	-	-	0.414
Baseline–120 min	6	0.032	−2	−1	0.549
Baseline–180 min	6	0.032	−2	−1	0.549
60 min–90 min	6	0.037	-	-	0.553
Flank region					
Baseline–20 min	6	0.03	−1.50004	−1	0.526
Baseline–30 min	6	0.032	−2	−1	0.526
Baseline–60 min	6	0.026	−2	−2	0.526
Baseline–90 min	6	0.026	−1	−1	0.526
Baseline–120 min	6	0.032	−2	−1	0.526
Baseline–180 min	6	0.026	−1	−1	0.526
Hind limb					
Baseline–20 min	6	0.034	−3	−1	0.558
Baseline–30 min	6	0.031	−3	−2.00004	0.558
Baseline–60 min	6	0.026	−3	−3	0.526
Baseline–90 min	6	0.032	−3	−2	0.558
Baseline–120 min	6	0.026	−3	−3	0.526
Baseline–180 min	6	0.034	−3	−2	0.558
Ataxia score					
Baseline–5 min	6	0.02	-	-	0.591
Baseline–10 min	6	0.03	−1.50004	−1	0.884
Baseline–20 min	6	0.034	−4	−2	0.938
Baseline–30 min	6	0.034	−4	−2.49999	0.938
Baseline–60 min	6	0.034	−4	−2.49999	0.938
Baseline–90 min	6	0.034	−4	−2.49999	0.938
Baseline–120 min	6	0.034	−4	−2.49999	0.938
Baseline–180 min	6	0.035	−3.5	−1.5	0.938
5 min–20 min	6	0.034	−3	−1	0.938
10 min–20 min	6	0.034	−2	−1	0.938

**Figure 4:**
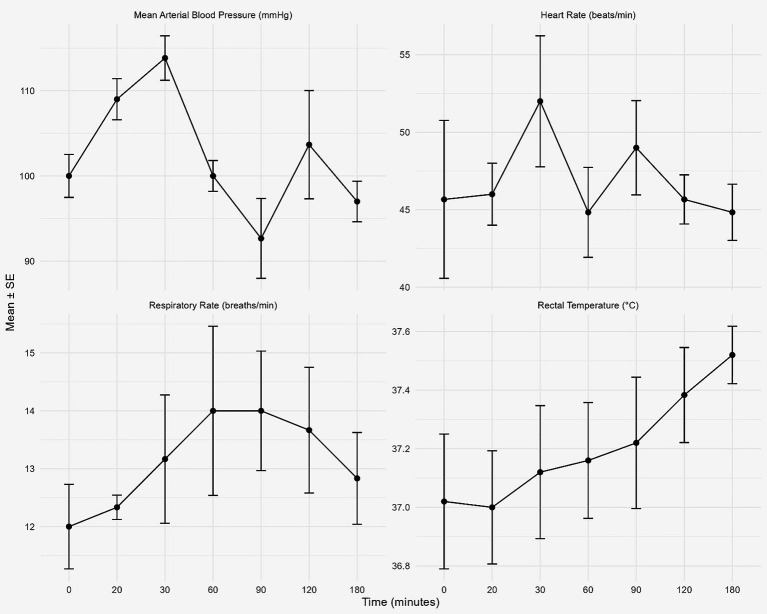
Illustrated cardiorespiratory parameters (mean ± SE) at the baseline and after epidural injection of articaine HCl with epinephrine in camels. Whereas heart rate (beat/min), respiratory rate (breath/min), mean arterial blood pressure (mmHg), and rectal temperature (°C) were registered at baseline, 20, 30, 60, 90, 120 and 180 min post epidural injection.

Comparison of cardiorespiratory parameters revealed significant differences only in mean arterial blood pressure and heart rate, with a moderate clinical effect observed in mean arterial blood pressure (size effect = 0.408). Rectal temperature and respiratory rate showed no statistically or clinically significant differences after lumbosacral epidural injection of articaine HCl 4% with epinephrine in camels (Table 4). Changes in heart rate and mean arterial blood pressure are described in Figure 4. However, after applying the Holm–Bonferroni correction, no significant differences in mean arterial blood pressure or heart rate were observed between time points (Table 5).

**Table 4:** ANOVA, effect size (generalized eta squared test), and descriptive statistics for cardiorespiratory variables after lumbosacral epidural injection of articaine HCl 4% with epinephrine in camels.

Parameter	MABP	HR	RR	RT
*N*	6	6	6	6
*p*-value	0.002	0.04	0.249	0.11
Generalized eta squared (*η*^2^G)	0.408	0.107	0.1	0.14
Descriptive statistics (mean ± SD)				
Baseline	100.16 ± 6.18	45.66 ± 10.48	12.00 ± 1.78	37.02 ± 0.56
20 min	108.00 ± 6.06	46.00 ± 4.89	12.50 ± 0.50	36.99 ± 0.47
30 min	114.00 ± 6.41	52.00 ± 10.35	13.16 ± 2.71	37.12 ± 0.56
60 min	101.00 ± 4.19	44.83 ± 7.11	14.00 ± 3.57	37.16 ± 0.48
90 min	94.67 ± 10.65	49.00 ± 7.46	14.00 ± 2.52	37.22 ± 0.55
120 min	107.50 ± 13.94	45.67 ± 3.88	13.66 ± 2.65	37.37 ± 0.39
180 min	97.00 ± 5.83	44.83 ± 4.44	12.83 ± 1.94	37.52 ± 0.24

MABP, mean arterial blood pressure; HR, heart rate; RR, respiratory rate; and RT, rectal temperature.

**Table 5:** Paired sample *t*-test after Holm–Bonferroni correction for cardiorespiratory variables with multiple comparisons at different time points pre and post lumbosacral epidural injection of articaine HCl 4% – epinephrine in camels (significant pairwise comparisons in regard to none adjusted *p-value* were only considered).

Mean arterial blood pressure (mmHg)	*N*	*t*-statistics	*p*-value	Confidence indicator 95% (low)	Confidence indicator 95% (high)	Holm–Bonferroni corrected *p*-value
Baseline–30 min	6	−3.01669	0.029	−25.621	−2.04566	0.472
Baseline–90 min	6	2.585553	0.049	0.042462	14.62421	0.736
20 min–180 min	6	3.782147	0.013	3.844055	20.15594	0.258
30 min–60 min	6	5.320076	0.003	7.149271	20.5174	0.066
30 min–90 min	6	3.536247	0.017	5.780112	36.55322	0.299
30 min–180 min	6	3.670907	0.014	5.045659	28.62101	0.274
90 min–120 min	6	−3.30662	0.021	−19.5515	−2.44855	0.362
Heart rate (beat/min)						
Baseline–30 min	6	−3.12358	0.026	−11.5454	−1.12125	0.522
20 min–90 min	6	−2.66557	0.045	−5.89309	−0.10691	0.79
30 min–60 min	6	3.057421	0.028	1.141163	13.19217	0.536
30 min–180 min	6	2.678101	0.044	0.287725	14.04561	0.79
60 min–90 min	6	−3.57143	0.016	−7.16568	−1.16765	0.336

## 4 Discussion

The present study investigated the anatomy of the lumbosacral junction in camels. The lumbosacral junction site consists of the skin, superficial fascia, multifidus lumborum muscle, supraspinous ligament, interspinous ligament, and ligamentum flavum, followed by the epidural space. A comparable anatomical arrangement has been reported in other species, including horses and calves (22, 23). In this study, the depth of the spinal cord from the skin in camels was 9.72 ± 0.58 cm. The dorsal length between the spinous processes of the seventh lumbar and first sacral vertebrae measured 9.32 ± 0.62 cm, whereas the ventral length at the level of the articular processes of these vertebrae was 4.33 ± 0.29 cm. Similar morphometric values have been reported in horses (24) and dogs (25, 26).

Notably, the depth of the epidural space in camels was greater than that reported in other domestic species. These findings provide important baseline anatomical data that directly support the safe and effective administration of epidural anesthesia in camels, representing the first documented measurements of epidural space depth in this species.

During anterior epidural anesthesia, cranial spread of the anesthetic agent may occur, potentially resulting in cardiopulmonary disturbances (7). In the present study, lumbosacral epidural injection resulted in minimal cranial spread of anesthesia. We hypothesized that this may be attributed to the cranial position of the hump, as well as the sternal recumbency position of the camels, which creates a slight ventral body inclination during drug administration and promotes caudal diffusion of the anesthetic agent under the influence of gravity. However, further investigations using dye infusion in the lumbosacral space are required to determine the extent of anesthetic diffusion. Moreover, in the present study, there is a hazard of accidental intrathecal injection during epidural anesthesia. Such misplacement can result in high spinal anesthesia, respiratory depression, and, in severe cases, apnea. Furthermore, it may induce marked hypotension and cardiovascular compromise. Therefore, strict attention to technique, dose adjustment, and aspiration before injection is essential to minimize these complications in veterinary epidural anesthesia (5, 15).

Articaine HCl (4%) is a relatively new local anesthetic agent in veterinary anesthesia and is classified as an intermediate-duration local anesthetic. Regarding the neurotoxic effect of articaine HCl, it may cause paraesthesia in humans due to its use in a high concentration (4%) than most other local anesthetics (27), which is difficult to determine in veterinary medicine. However, identification of behavioral changes such as intense licking, biting at the injection site, crying, and animal signs of skin itch were not observed after lumbosacral epidural injection in the present study. Regarding articaine toxicity, previous studies revealed that repeated subcutaneous administration of articaine HCl in dogs had no pathomorphological systemic changes even though it was received at systemically toxic doses. The no-effect level (NOEL) for articaine HCl was 40 mg/kg/day (28). Moreover, as hypothesized previously, permanent neurotoxicity after a single application of articaine HCl to healthy nervous tissue is, fortunately, a rare complication clinically (13).

Owing to the lack of published data on epidural analgesia using articaine HCl in camels, the findings of the present study were compared with previous studies employing lidocaine HCl for epidural anesthesia in this species, as both agents belong to the same pharmacological class of local anesthetics. Previous studies have reported that the onset of analgesia following the first intercoccygeal epidural injection of lidocaine in camels was 6.5 ± 2.3 min (20), whereas the onset of perineal analgesia after lidocaine epidural injection at the same site was 9.25 ± 1.25 min (8). The results of the present study are consistent with these findings, as the onset of perineal analgesia following lumbosacral epidural administration of 4% articaine HCl with epinephrine was 7.6 ± 2.5 min. Conversely, one study reported a more rapid onset of analgesia (3.67 ± 0.33 min) following sacrococcygeal epidural injection of lidocaine in camels (9). This finding closely parallels the onset of analgesia observed in the tail region in the present study (4.6 ± 0.6 min) after lumbosacral epidural injection of 4% articaine HCl.

In the present study, the onset of analgesia was evaluated separately for each anatomical region. The fastest onset of analgesia was observed in the tail within 4.6 ± 0.6 min, followed sequentially by the hind limbs, perineal, inguinal, and umbilical regions, whereas the flank region exhibited the slowest onset of analgesia (10.0 ± 0.5 min). Variations in onset times among different regions may be attributed to differences in nerve size, the anatomical location of nerves within the spinal canal, differential distribution of the anesthetic agent within the epidural space, and the degree of nerve myelination (2, 6, 16, 29).

The rapid onset of tail analgesia observed in this study may also be related to the pharmacological properties of articaine HCl. Articaine contains a thiophene ring, which enhances lipid solubility and facilitates diffusion across lipid-rich nerve membranes, allowing rapid access to target receptors (13).

Based on prior literature and a pilot study conducted before the present experiment, the pin-prick method was found to have limited value for assessing analgesia in camels. Camels possess a remarkable ability to tolerate pain with minimal outward signs of distress, making pain recognition and scoring challenging in this species (9). Conversely, skin pinching using towel forceps provided more reliable and reproducible analgesic assessment in the present study.

Analgesic scoring revealed mild to moderate analgesia (median score 1–2) in all examined regions except the hind limbs; moderate analgesia in the inguinal region (median score 2–2.5); and moderately deep analgesia (median score 2–3) was consistently observed. This finding is consistent with previous reports indicating that epidural blockade at the lumbosacral level produces dense sensory blockade of the pelvic limbs, often resulting in deep analgesia, while more cranial regions may exhibit lower degrees of sensory blockade (30).

Differences in the duration of analgesia among body regions were also observed, with analgesia persisting for 180 ± 48.9 min in the tail, 195 ± 57.4 min in the umbilical region, 225 ± 57.4 min in the perineal region, 240 ± 48.9 min in the inguinal region, and up to 255 ± 90 min in the hind limbs. This contrasts with previous literature describing the quick hydrolysis of articaine HCl by esterases, shortening its duration of action (13). This prolonged duration of analgesia may be attributed to the vasoconstrictive effect of epinephrine, which reduces systemic absorption and prolongs the local anesthetic action (2, 31).

Ataxia resulting from motor blockade of the spinal nerves was observed at 8.6 ± 2.7 min following lumbosacral epidural injection. Camels subsequently became recumbent at 21.0 ± 3.5 min post-injection. Only one of the six camels exhibited ataxia without progressing to recumbency. A more intense locomotor blockade, characterized by earlier onset and prolonged duration of recumbency, was observed in two pregnant female camels. Overall, the total duration of recumbency following epidural administration of 4% articaine HCl was 203 ± 19.8 min.

Conversely, recumbency has not been reported in previous studies involving the first intercoccygeal epidural injection of lidocaine HCl in camels (8, 20, 32). The severe ataxia and recumbency observed in the present study (locomotor score 3–3.5) approximately 20 min after lumbosacral epidural injection may be explained by several factors. First, the lumbosacral epidural approach directly affects pelvic-limb innervation due to its proximity to the lumbosacral plexus, resulting in a more pronounced motor block than sacrococcygeal or intercoccygeal epidural techniques (2, 16). Second, articaine HCl exhibits greater lipophilicity than lidocaine HCl, facilitating enhanced diffusion across nerve membranes and producing a more intense motor blockade (13). Third, pregnancy may predispose animals to recumbency during epidural analgesia due to increased abdominal mass, altered weight distribution, and augmented motor blockade, particularly in advanced gestation (2, 16). However, further investigation with a larger sample size of pregnant camels is recommended to study the correlation between pregnancy and time of recumbency after lumbosacral epidural analgesia in camels.

The prolonged recumbency observed in the present study may also be attributed to the addition of epinephrine, which reduces systemic absorption and prolongs the anesthetic effect (33, 34). In the present study, prolonged recumbency was a notable complication following epidural anesthesia. Extended recumbency may result in muscle ischemia, pressure-induced myopathy, and peripheral nerve compression, which can delay recovery and impair the animal’s ability to stand again. Additionally, recumbency adversely affects respiratory function, especially in lateral positions, leading to reduced lung expansion and potential hypoventilation. Fortunately, lateral recumbency was not observed in the present study after lumbosacral epidural injection. Furthermore, sustained sympathetic blockade may contribute to hypotension and reduced tissue perfusion, exacerbating systemic compromise. All examined camels in the present study stood again after the end of anesthesia without any observed complications. In general, difficulty in regaining a standing position is a critical welfare and clinical concern in camel species, as it may lead to trauma and prolonged recovery (16).

Minimal alterations in cardiorespiratory variables were observed in the present study. No significant changes were detected in rectal temperature or respiratory rate following lumbosacral epidural injection of 4% articaine HCl with epinephrine. However, maximal alterations in cardiorespiratory parameters were observed in mean arterial blood pressure post-injection. Similarly, another study reported significant increases in blood pressure following administration of lidocaine combined with epinephrine compared with baseline values (35). A slight but significant decrease in mean arterial blood pressure was recorded at 90 min after epidural analgesia, a finding consistent with previous observations in camels (36). Importantly, all recorded heart rate and blood pressure values in the present study remained within the normal physiological ranges reported for camels. Authors documented a normal heart rate of approximately 50 beats per minute and arterial blood pressure values ranging from 76 to 115 mmHg in healthy camels (37). Therefore, these minimal cardiorespiratory changes should be interpreted as statistically rather than clinically significant. Moreover, it is important to distinguish between statistical significance and clinical relevance, as the alterations detected in this study do not appear to adversely affect the animal’s physiological status. However, after Holm–Bonferroni corrections, no significant changes were recorded in any cardiorespiratory parameters in the present study.

Limitations of this study include a lack of homogeneity among animals, as two pregnant camels were used, which may have different epidural space pressures and different motor block intensities. Furthermore, injection into the lumbosacral space in camels conveys hazards of accidental subarachnoid injection, which requires more caution and precise injection. Although no neurotoxic effect or paraesthesia was observed in this study after epidural injection, long-term neurological follow-up in camels after lumbosacral epidural injection is required in further studies to assess the possible neurotoxic effect of articaine HCl. Furthermore, the lack of surgical procedures to assess anesthetic effects under clinical conditions is considered a limitation of the present study.

## 5 Conclusion

The lumbosacral epidural space in camels can be easily and reliably identified using consistent anatomical landmarks. Preliminary findings of this study suggested that lumbosacral epidural administration of articaine HCl 4% with epinephrine provided a rapid onset of analgesia, producing mild to moderate analgesia in the tail, perineum, inguinal, flank, and umbilical regions and moderately deep analgesia of the hind limbs, accompanied by a prolonged recumbency without definite changes in cardiorespiratory parameters. These preliminary findings suggested that lumbosacral epidural injection of articaine HCl with epinephrine can be used as an alternative technique for achieving extensive epidural analgesia in camels. However, further studies are recommended to investigate higher doses of articaine or even to evaluate multimodal lumbosacral epidural protocols combining articaine with opioids or α2-adrenergic agonists to optimize analgesic efficacy and motor effects.

## Data Availability

The raw data supporting the conclusions of this article will be made available by the authors, without undue reservation.
